# Numerical investigation of high-temperature proton exchange membrane fuel cell conductivity at different parameters

**DOI:** 10.1038/s41598-025-89277-6

**Published:** 2025-02-13

**Authors:** A. Samir, M. S. Maowwad, M. A. Farahat, M. Talaat

**Affiliations:** 1 Electrical Power and Machines Department, Faculty of Engineering, Zagazig University, P.O. 44519, Zagazig, Egypt; 2 Manufacturing and workshops administration, Arab Contractors Company, Cairo, Egypt; 3 Faculty of Engineering and Technology, Egyptian Chinese University, P.O. 11787, Cairo, Egypt

**Keywords:** HTPEMFC, 3D simulation, Membrane effect, Finite element method, COMSOL Multiphysics, Engineering, Energy science and technology, Fuel cells

## Abstract

This study uses the finite element technique to analyse a multi-dimensional model for a polyelectrolyte membrane fuel cell at high working temperature. A computational fluid dynamics (CFD) technique implements and solves this model. In addition, the membrane’s thickness, and catalyst layer’s thickness parameters have been studied. Membrane thickness is varied from to and the length of the fuel cell from to. The performance of the fuel cell was studied, analysed, and discussed for each case using the polarization curves and output power. The results indicate that the performance of fuel cells is enhanced by a thinner membrane than a thicker one with an increase in loading. The performance is approximated at light loads. Furthermore, the concentration of water at the cathode side of the fuel cell is highly affected by the change in fuel cell length more than the thickness of the membrane. Comparative analysis with prior research demonstrates strong agreement with our consequences.

## Introduction

**Fig. 1:**
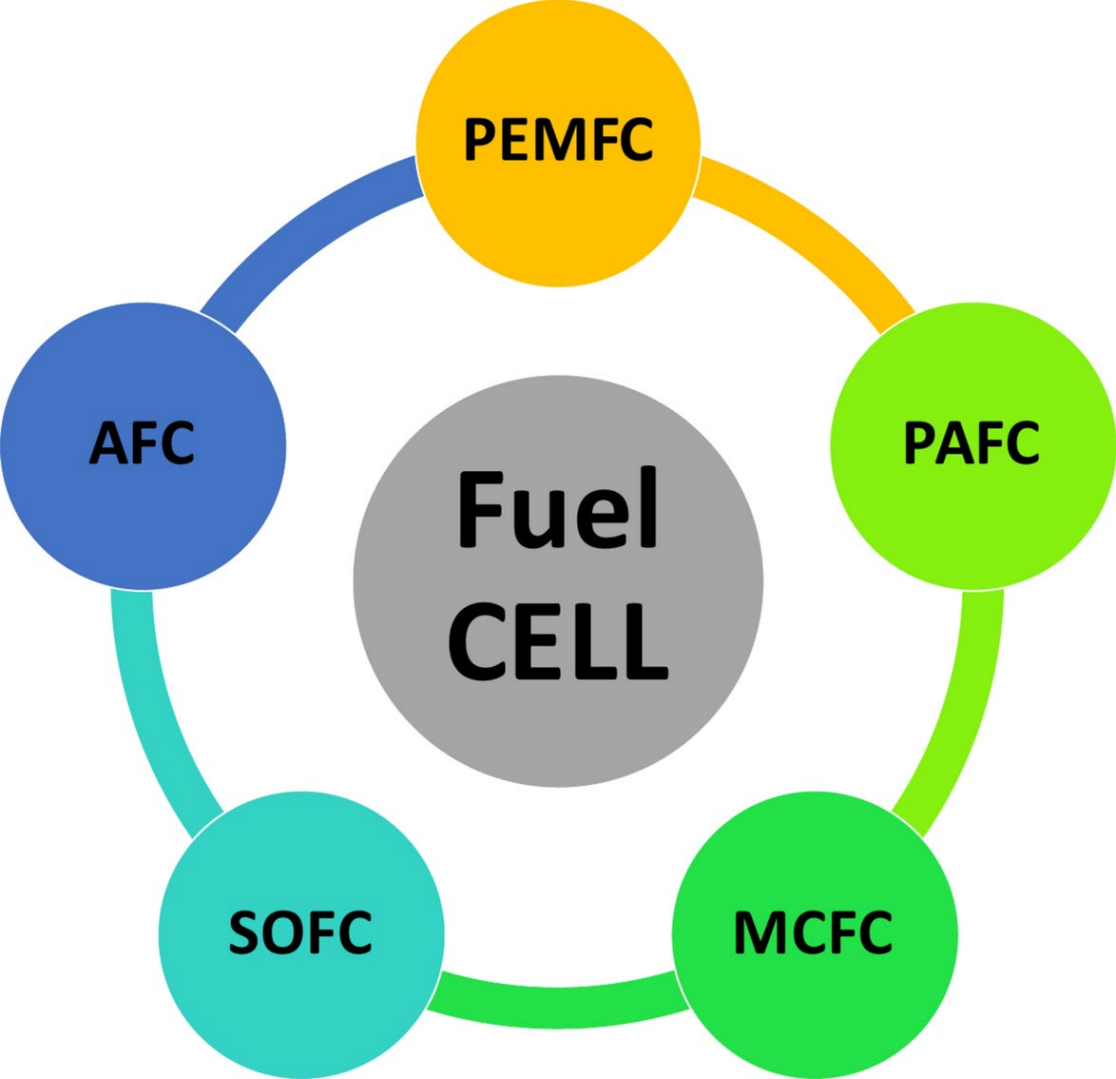
Different types of fuel cell.

According to the transition of the energy structure from natural and coal energy to the dominance of oil and gas in the twentieth century and continues to present day, becoming an indispensable source of energy in industry and daily life^1,2^. With the growing significance of energy security and environmental pollution, it is necessary to develop the energy structure and find cleaner and non-polluting alternatives^3^. Hydrogen energy, being abundant and environmentally friendly, offers a promising solution^4^. Furthermore, hydrogen’s energy density (approximately 120 MJ/kg) is more than double that of natural gas and crude oil^5^. Fuel cell technology constitutes a pivotal domain within the spectrum of hydrogen energy applications, offering compelling advantages such as environmental sustainability, energy efficiency, and rapid system response^6^. As shown in Fig. 1, fuel cells can be categorized based on their electrolyte, resulting in distinct types such as alkaline fuel cells (AFC)^7^, solid oxide fuel cells (SOFC)^8,9^, phosphoric acid fuel cells (PAFC)^10^, molten carbonate fuel cells (MCFC)^11^, and proton exchange membrane fuel cells (PEMFC)^12,13^. The last type exhibits superior performance characteristics, including high peak efficiency, high power output, and low operating temperature. Therefore, PEMFC has revealed more application prospects in various clusters, such as the maritime and automotive industries, which are the most distinguished and promising^6,14^. It was reported in^14^ that the number of fuel cell vehicles increased to 51,437 units at the end of 2021 with an extensive growth rate of 48%, exposing the great market demand of PEMFC.

**Fig. 2:**
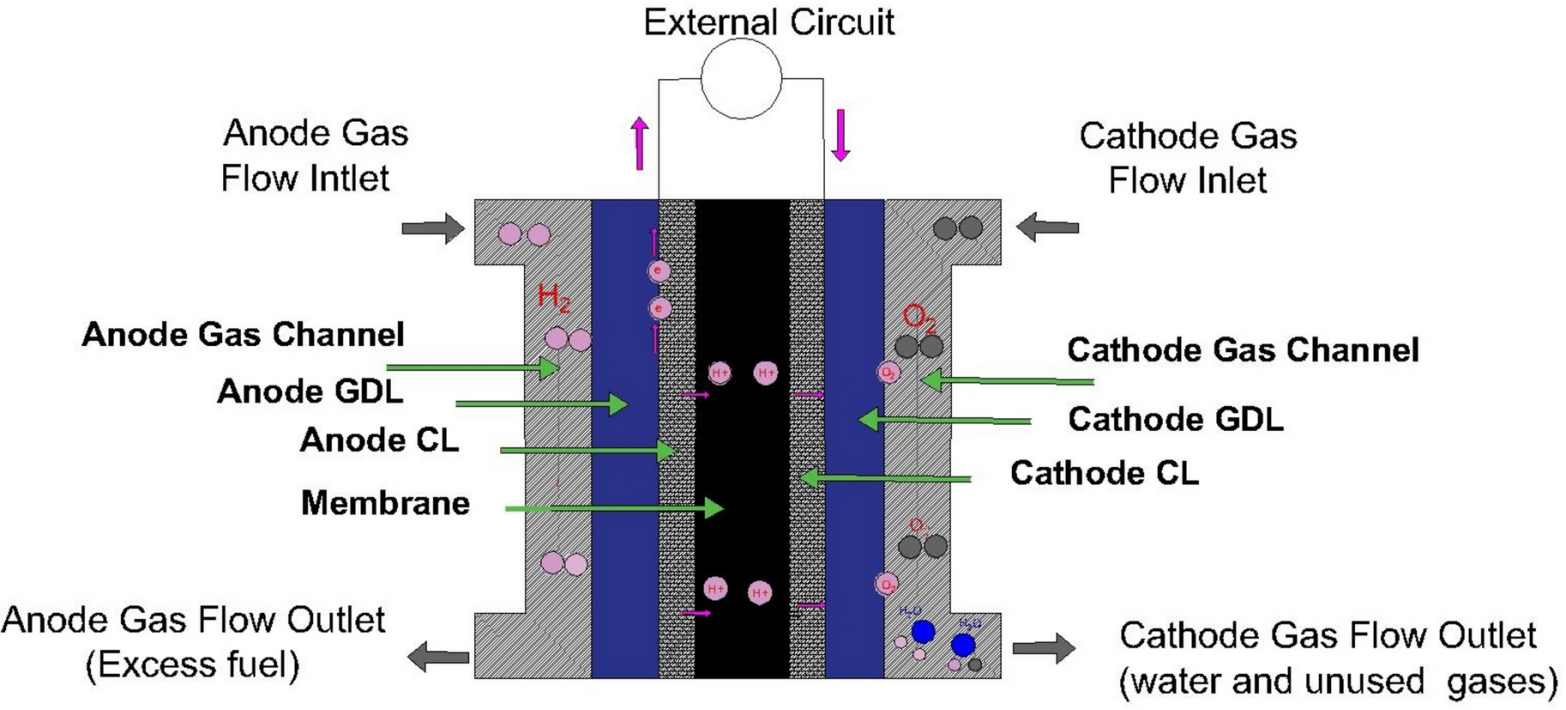
Schematic illustration of the structure of fuel cell.

PEMFC unit is mainly constructed from multiple cells to fulfill the required voltage and power. As shown in Fig. 2, a typical cell comprises a multi-layered architecture encompassing a bipolar plate (BP), catalyst layer (CL), proton exchange membrane (PEM), gas diffusion layer (GDL), and microporous layer (ML)^15^. At the anode side, hydrogen undergoes an oxidation reaction, which releases electrons transferred to the cathode throughout an external circuit. Concurrently, protons migrate across the polymeric membrane towards the cathode^16^. At this cathode, oxygen bonds with electrons, and protons in a reduction reaction, to yield water as a byproduct. Consequently, the whole cell transforms the chemical energy inherent within the hydrogen–oxygen system into electrical and thermal energy^17^.

Now, the PEMFC technology has attained a degree of maturity. However, its application is still restricted due to high cost and durability limitations^18^. Relating to the International Energy Agency Report 2024^19^, there is a gap between the industrialized cost and FC cost for each kW, where its average is (90$/kW) still high compared to the ideal industrialized cost^20^. Remarkably, for fuel cell electric vehicles (FCEVs), the PEMFC system constitutes a substantial portion of the overall vehicle cost, accounting for approximately two thirds^4,21^.

PEMFCs performance can be studied using either experimental methods or mathematical modeling. However, experimental investigations inherently entail significant challenges. These limitations are inherently time-consuming, present challenges in ensuring efficient hydrogen supply and storage infrastructure, and necessitate substantial upfront capital investment^22^. Conversely, mathematical modeling offers a more cost-effective approach to PEMFC performance evaluation. Furthermore, the development of a precise mathematical model provides a valuable tool for assessing the system feasibility prior to its physical realization^23^. Developing accurate mathematical models is essential for the successful design and testing of PEMFCs. Accurate determination of model parameters is crucial for the development of reliable PEMFC models. However, these parameter values are frequently not readily available from manufacturers, demanding a careful and systematic identification process^22^. These include operating conditions such as temperature, pressure, and reactant gas flow rates^24,25^. Furthermore, material properties play a crucial role, comprising the proton conductivity of the membrane, the electrocatalytic activity of the electrodes, and the gas transport properties of the diffusion layers^26,27^. Additionally, geometric factors such as cell thickness, electrode area, and channel geometry significantly influence the overall performance and efficiency of the PEMFC^28,29^.

The membrane should have a permeability property that permits the protons to pass through it and reject the electrons. The membrane of PEM is located between two layers of anode and cathode sides. The fuel (Hydrogen) is always fed to the cell from the anode side, and air (oxygen) is fed to the other side^30^. Each catalyst layer facilitates the reaction as shown in Fig. 2, two main reactions happen inside the cell^31^. At the anode side, the first reaction is called Hydrogen oxidation or reduction, where Hydrogen is decomposed into positive ions and negative charges according to Eq. (1). The membrane acts as a barrier for electrons, but it allows positive ions to travel from the anode to the cathode.

On the other hand, the second reaction is at the cathode side where air or oxygen and the ions reunify for the stability of the system and form oxygen and water vapor Eq. (2)^32,33^. Free electrons pass in the external circuit outside the membrane from the anode to the cathode side by producing the electric current needed from the cell and heat release from the reaction as demonstrated in Eq. (3).

Reaction at anode side:

1 $$H_{2} \to 2H^{ + } + 2e^{ - } ,$$

Reaction at cathode side:

2 $$\frac{1}{2}{\text{O}}_{2}+2{H}^{+}+2{e}^{-}\to {H}_{2}O ,$$

Then, whole reaction of cell:

3 $${H}_{2}+\frac{1}{2}{\text{O}}_{2}\to {H}_{2}O+heat .$$

Accordance to operating temperature can be characterized by splitting the fuel cells into two types^34–36^. Firstly, the low-temperature type depends on the Nafion membrane, where the operating temperature has a value between room temperature to 100 °C. Low-temperature fuel cells, especially PEM fuel cells, exhibit high power-to-weight ratios, high response, high electrical efficiency, and reduced auxiliary system complexity. These characteristics are consistent with the critical demands of automotive propulsion, making them the leading technological selection for this large-scale market. In contrast, low-temperature conditions necessitate the employment of expensive platinum group metal catalysts and constrain fuel options to primarily hydrogen and methanol^37^.

Secondly, high-temperature PEM fuel cells (HTPEMFCs), have involved significant attraction due to their ability to handle impurities, allowing the use of gas mixtures with various hydrocarbons without needing prior purification^38^. Furthermore, HTPEMFCs, which can operate to approximate $160 ^\circ \text{C}$, exhibit high thermal energy^39^. This energy is easily controlled for water and thermal applications^30,40,41^. The membrane is made from polybenzimidazole (PBI), where the operating temperature can go up to a value up to 200 °C^42,43^. The fuel cell produces electricity directly through electrochemical reactions. Although these reactions are similar to traditional batteries. Fuel cells can generate electrical energy providing the fuel feeds them, without recharging solving the problem of traditional batteries^44,45^.

In this paper, a model is created in the COMSOL Multiphysics program^31^ and different cases of study are used for simulating the fuel cell of PEM. The membrane thickness and the length of the fuel cell are obtained as variable parameters for the study of the fuel cell performance. These parameters can be altered during the design stage of the fuel cell and are effective in the fuel cell selection. The cell performance is studied by maintaining the change of the current density at the end of the anode side. The finite element method (FEM) is used to model the fuel cell.

## Modelling

### Model assumption

Due to the high temperature and pressure conditions in the HTPEMFC, water is exclusively in a gaseous or vapor state^46^. In addition to its unique properties, PBI membranes have a negligible water drag coefficient, unlike Nafion membranes used in low-temperature PEMFCs^47^. The proton transfer mechanism in the PBI membrane is mediated by the phosphoric acid incorporated within it^48^. The behavior of the gas mixture is assumed to be ideal, and due to the slow flow rate, the gas moves in a smooth-layered pattern. The layer of gas diffusion is made of a porous material that has the same properties throughout.

In recent studies, most devices and cases are modeled using the finite element method (FEM) that is used in several fields of medicine, electricity, electronics, and chemistry. The main idea of the finite element method is that the surface of the materials is divided into small finite parts and then it is analyzed using the laws of electricity, physics, and chemistry. Based on this, fundamental equations that govern fuel cell operation are illustrated in the first principle-based in the incoming subsections.

### Navier–Stokes equation

Gas flux is considered to be a laminar and incompressible type. Based on this, the Navier–Stokes equation is used in gas flow channels to describe and calculate the mass and momentum transfer of the fluid in the HTPEMFC^49,50^. Conservation of mass and momentum are given by Eqns. (4) and (5), respectively.

4 $$\nabla .u=\frac{Q}{\rho } ,$$

5 $$\rho u.\nabla u=\nabla \left(-PI+\mu \left[\nabla u+{\left(\nabla u\right)}^{T}\right]\right) ,$$

where, $u$ is the velocity vector of fluid mixture ($\text{m}/\text{s}$), $Q$ is the mixture mass $(\text{kg}/{\text{m}}^{3}.\text{s})$, $\rho$ is the mixture density $(\text{kg}/{\text{m}}^{3})$, and $P$ is the pressure ($\text{N}/{\text{m}}^{2}$). As, $\mu$ is the dynamic viscosity of the mixture $\text{kg}/(\text{m}.\text{s})$ and is calculated as:

6 $$\mu =\sum_{i}{x}_{i}.{\mu }_{i}$$

where, $x$ is the molar fraction and the subscript $i$ refer to different species.

### Darcy’s law

Due to the gas diffusion layer manufactured from isotropic and homogeneous porous material, Darcy’s law is used to define momentum transfer in a porous medium. Therefore, the generation part of the flow is necessary for insertion into the Navier–Stokes according to the following equations.

7 $$Q=-\frac{k}{\mu } \nabla P,$$

8 $$\rho \left(u.\nabla \right)u+\nabla P-\nabla .\mu \left[\nabla u+{\left(\nabla u\right)}^{T}\right]=-\frac{\mu }{k}u,$$

where, $k$: is the permeability of the GDL (m^2^).

### Maxwell–Stefan equation

This equation explains the diffusion phenomena in a multi-layer system consisting of gaseous and liquid mixtures, use to calculate the concentration of various chemicals in solutions. It also models chemical reactions and the movement of chemicals through diffusion, convection, and migration in both concentrated and dilute solutions.

The movement of concentrated species plan is used to analyze gas and liquid mixtures where the concentrations of different chemicals are difficult to identify as a solvent and similar quantity. The mixture properties rely on the fluid components and interactions between all the chemicals. Separately, this occurs for the anode and cathode GDL domains, as well as a linear mass fraction for both layers.

For laminar flow in the porous GDL, the pressure drop is directly proportional to the velocity of gas. This relationship is calculated by:

9 $$\left(\mu /k\right)u=\nabla \left\{-PI+\left(\mu /\varepsilon \right)\left[\nabla u+{\left(\nabla u\right)}^{T}\right]\right\},$$

where, $\varepsilon$ and $k$ are the porosity and permeability of the GDL, respectively.

Relating species conservation principle,

The movement of different gases throughout the entire computational domain is described by the Maxwell–Stefan equation. This equation calculates the flow of each gas based on its mass fraction. So, Eq. (10) presents the general formula of the Maxwell–Stefan equation^51^.

10 $$\nabla \left\{-\rho {w}_{i}\sum_{j=1}^{N}{D}_{ij}\left[\frac{M}{{M}_{j}}\left(\nabla {w}_{j}+{w}_{j}\frac{\nabla M}{{M}_{j}}\right)+\left({x}_{j-}{w}_{j}\right)\frac{\nabla P}{P}\right]+\rho {w}_{i}u\right\}={R}_{i}$$

where ${D}_{ij}$ is the coefficient of binary diffusion^52^, $x$ is the ratio of molar, $w$ is the mass ratio, $M$ is the mass of molecular, $R$ is the universal gas constant $(8.314 \text{J}/\text{mol}.\text{K})$, and $T$ is temperature $(\text{K})$. In addition, $\rho$ is the density of gas mixture given by:

11 $$\rho =\frac{\left(\sum_{i}{x}_{i}\cdot {M}_{i}\right)P}{R\cdot T}$$

Different species are represented by the subscripts $i$ and $j$. Reaction rate ${R}_{i}$, is a measure of how much reactant is consumed and how much product is produced during electrochemical reactions in the catalyst layer. ${R}_{i}$ was computed as^53^:

12 $${R}_{{H}_{2}}=-\frac{{j}_{a}}{2F}{M}_{{H}_{2}}, {R}_{{O}_{2}}=-\frac{\mid {j}_{c}\mid }{4F}{M}_{{O}_{2}}, {R}_{{H}_{2}O}=\frac{\mid {j}_{c}\mid }{2F}{M}_{{H}_{2}O}$$

Just oxygen and water are solved on the cathode side because the third species is always obtainable using the mass balance equation, which is as follows:

13 $${w}_{N2}=1-{w}_{{O}_{2}}-{w}_{{H}_{2}O}$$

Hydrogen is solved on the anode side, and the mass fraction of water is obtained as follows;

14 $${w}_{{H}_{2}O}=1-{w}_{{H}_{2}}$$

The empirical correlation can be used to compute the binary diffusivities ${D}_{ij}$ in the Maxwell–Stefan equation as:

15 $${D}_{ij}={D}_{i{j}_{0}}{(\frac{T}{{T}_{0}})}^{1.5}$$

where ${D}_{i{j}_{0}}$ is the reference binary diffusivity and ${T}_{0}$ is the reference temperature. The effective binary diffusivity in the porous media is modified to take into consideration the impact of the porous GDL’s porosity:

16 $${D}_{i\text{j}}^{\text{eff}}={D}_{i\text{j}}{(\varepsilon )}^{1.5}$$

### Butler–Volmer Formula

In the catalyst layers, the electrochemical reaction is illustrated by the Butler-Volmer equation. The following equation describes the continuity of current in a conducting medium for the current transport^54^:

17 $$j={j}_{0}\left({e}^{\alpha nF\eta /\left(RT\right)}-{e}^{-\left(1-\alpha \right)nF\eta /\left(RT\right)}\right)$$

where, ${j}_{0}$ is the exchange current density, $n$ is the electron number of transfer in the electrochemical reaction, $\alpha$ is the charge transfer coefficient, $F$ is the Faraday constant, and $\eta$ is the over-potential.

Ionic current and electronic current are the two types of current that can be generated in a PEMFC relating to the charge conservation law. An electronic current is produced when electrons only go through the solid matrix of electrodes, whereas protons move through the ionic conductor membrane to form an ionic current.

Ohm’s law is applied to obtain the current continuity equations^55^:

18 $$\nabla .\left( { - \sigma_{s} \nabla .\emptyset_{s} } \right) = S_{s}$$

19 $$\nabla .\left( { - \sigma_{m} \nabla .\emptyset_{m} } \right) = S_{m}$$

where, $S$ is the current source term $(A.{m}^{-3})$, $\sigma$: is the effective electric conductivity $(S.{m}^{-1}),$, and the subscript (s): indicates the solid phase property while (m) indicates the membrane property.

The electrochemical reaction, which only takes place in the catalyst layers of the anode and cathode sides, produces the source terms in the electron and proton transport equations. The anode catalyst layer is given as follows:

20 $$\left\{\begin{array}{c}anode catalyst layer \left\{\begin{array}{c}{S}_{m}={j}_{a}\\ {S}_{s}={-j}_{a}\end{array}\right.\\ cathode catalyst layer \left\{\begin{array}{c}{S}_{m}={j}_{c}\\ {S}_{s}={-j}_{c}\end{array}\right.\end{array}\right.$$

where, the electrochemical reactions at two parts catalyst layers is represented by the transfer current density, ${j}_{a}$ and ${j}_{c}$, respectively.

The transfer current density of the anode (${j}_{a}$) and cathode (${j}_{c}$) is connected to the source terms in both the species and charge equations. This density is determined by the mitigated Butler-Volmer formula, which is provided as:

21 $${j}_{a}=a{i}_{0,a}^{ref}{\left(\frac{{C}_{{H}_{2}}}{{C}_{{H}_{2}ref}}\right)}^{0.5}(\frac{{\alpha }_{a}+{\alpha }_{c}}{RT}F{\eta }_{a})$$

22 $${j}_{c}=a{i}_{0,c}^{ref}\left(\frac{{C}_{{O}_{2}}}{{C}_{{O}_{2}ref}}\right)exp\left(\frac{{-\alpha }_{c}}{RT}F{\eta }_{c}\right)$$

where the potential difference between the electrolyte and solid matrix is represented by ${\eta }_{a,c}$, which is defined as:

23 $$\left\{ {\begin{array}{*{20}c} {\eta_{a} = \emptyset_{s} - \emptyset_{e} {\text{at anode side}}} \\ {\eta_{c} = \emptyset_{s} - \emptyset_{e} - U_{oc} {\text{at cathode side}}} \\ \end{array} } \right.$$

Stoichiometric coefficient, electrode surface area, and flow channel geometry are used to determine the inlet gas velocity, which is defined as follows:

24 $${{U}_{in\_cathode}=\lambda }_{c}\frac{1}{4F}{x}_{{o}_{2}}RT/(P.{A}_{channel}.{n}_{channel})$$

25 $${{U}_{in\_anode}=\lambda }_{a}\frac{1}{2F}{x}_{{H}_{2}}RT/(P.{A}_{channel}.{n}_{channel})$$

where, ${n}_{channel}$ and ${A}_{channel}$ are the number and cross-sectional area, respectively. Also, ${U}_{in\_\_cathode}$ and ${U}_{in\_anode}$ are, respectively, the means of inlet velocities on the cathode and anode sides.

Based on the humidified air and associated temperature, the species fraction at the inlet is computed for the boundary conditions. The back pressure at the flow channel’s output is adjusted to equal atmospheric pressure. It is presumed that the flow is complete. The condition of a no-slip boundary is employed for additional impermeable types of walls and surfaces, and the boundary in the land area’s central plane is set symmetrically. The fuel cell operating voltage is equal to the value of the cathode current collector, while the anode current collector is set to 0 V. According to Table 1, the remaining boundaries are symmetrical or insulated. All these equations are solved by computer programs to get the required results.

**Table 1:** The geometric dimension parameters and some initial values of the 3D model of the fuel cell model cases study.

Parameter	Value
Length of cell	$0.02\stackrel{to}{\to }0.05 \text{m}$
Porous electrode thickness	$20 \mu \text{m}$
Membrane thickness	$(20\stackrel{to}{\to } 120) \mu \text{m}$
GDL porosity	$0.4$
GDL permeability	$1.18\times {10}^{-11} {\text{m}}^{2}$
GDL electric conductivity	$222\text{ S}.{\text{m}}^{-1}$
Inlet ${H}_{2}$ mass fraction	$0.743$
Inlet ${H}_{2}O$ mass fraction	$0.023$
Inlet oxygen mass fraction	$0.228$
Anode inlet flow velocity	$0.2 \text{m}.{\text{s}}^{-1}$
Cathode inlet flow velocity	$0.5 \text{m}.{\text{s}}^{-1}$
Viscosity of anode	$1.19\times {10}^{-5} \text{Pa}\cdot \text{s}$
Viscosity of cathode	$2.46\times {10}^{-5}\text{ Pa}\cdot \text{s}$
Temperature	$453 K$
Voltage	$0.9 \text{V}$
Conductivity of membrane	$9.825 \text{S}.{\text{m}}^{-1}$
Pressure	$101.33 \text{kPa}$

## Simulation model

### Models’ description

**Fig. 3:**
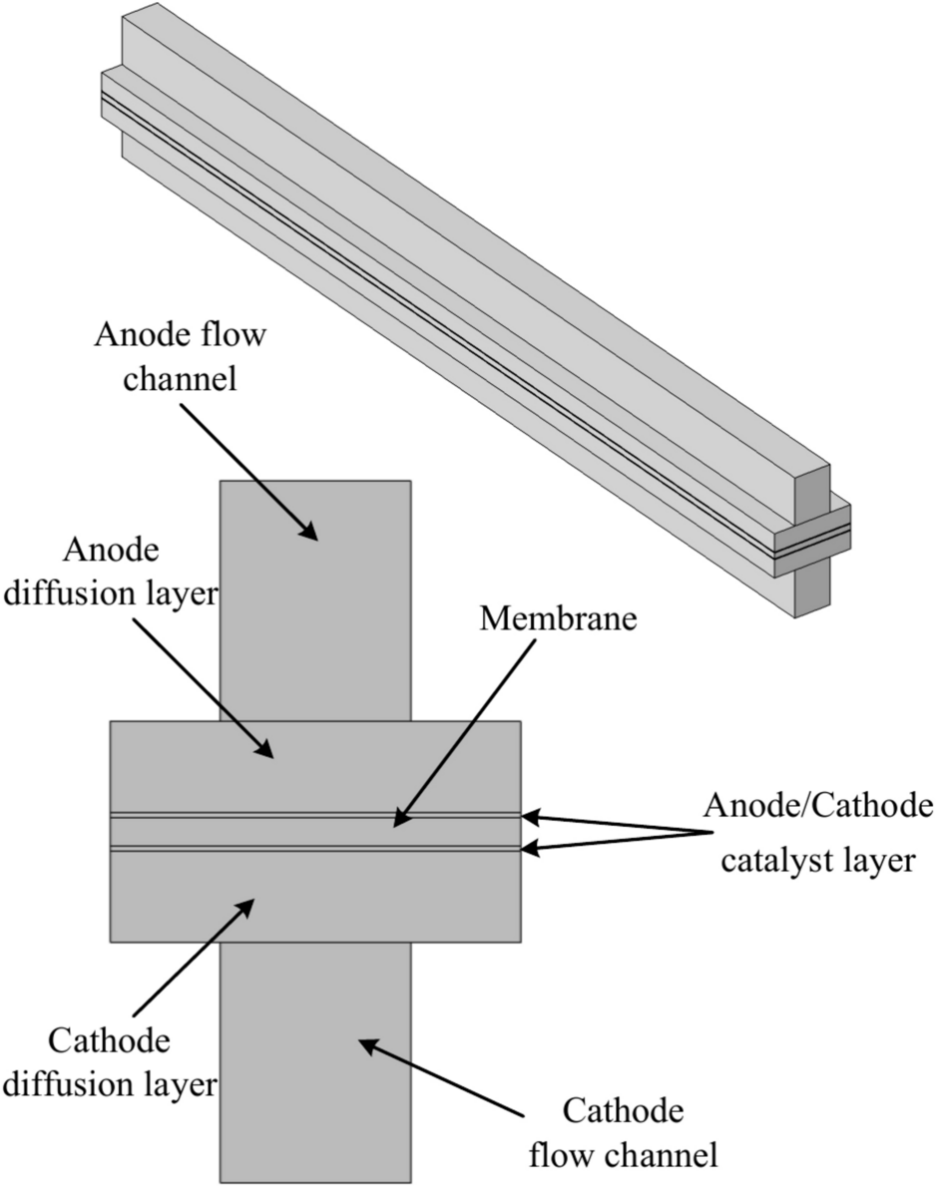
A schematic diagram of the 3D- model geometry of HT PEMFC.

This mode presents different cases of study where all cases have the same geometry in the arrangement of the layers, where each model consists of three main regions called the anode and cathode sides, as well as the membrane, as shown in Fig. 3.

Each region consists of sub-regions. The anode side region consists of the anode flow channel, anode gas diffusion layer, and anode catalyst layer. The cathode side region involves the flow channel, gas diffusion layer, catalyst layer, and membrane.

**Fig. 4:**
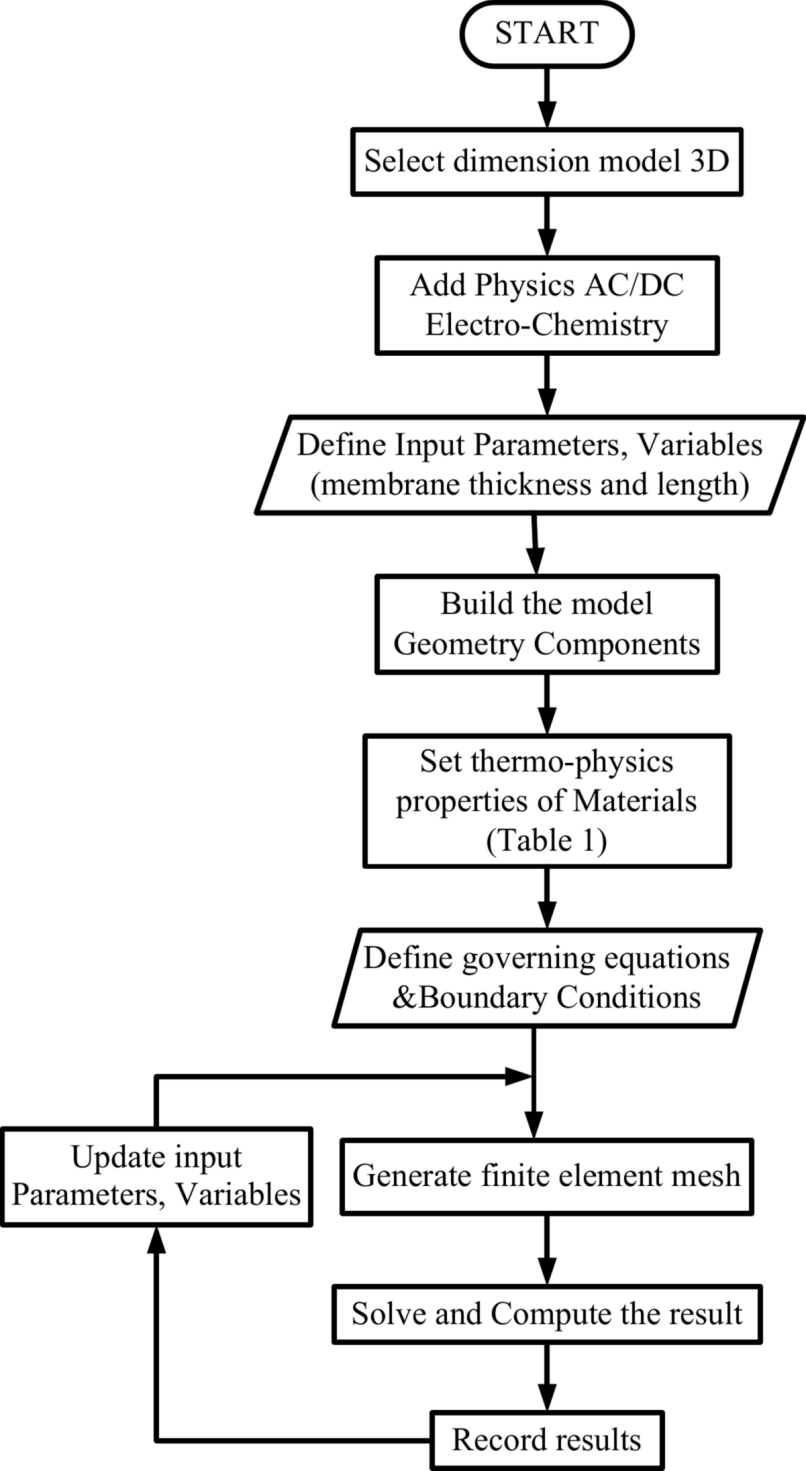
Flowchart illustrates the proposed methodology in simulation.

For studying the characteristics of the fuel cell and noticing the system performance due to the change in the dimensions of the model, the thickness of the membrane varied with different values $(20, 40, 60, 80, 100 \& 120)$ μm and the length of the fuel cell varied with equal different values from $0.02$ to $0.05\text{ m}$. At each value of the fuel cell length, the cases are set for each value of membrane thickness, and study is done for each case. The parameters definition and their values are described in Table 1. In addition, Fig. 4 reviews the steps of the simulation model in a flowchart form.

The following boundary conditions and assumptions are applied to the simulation model in order to simplify it and decrease the amount of computation with adequate accuracy.

The following are the underlying presumptions:

- homogenous and isotropic porous media.
- It’s the ideal gas.
- There is disregard for the contact resistance.
- A laminar gas flow occurs.
- The model is used in a stationary operation state.

Boundary conditions for study are:

- For all the walls in the channel, there is no slip boundary condition.
- Every wall in the channel has no slip boundary condition.
- Initial values are set to be zero.
- At the channel’s outlet, backpressure is absent.
- Both the inlet and the output have their outer margins set to zero.
- Bipolar plates connected to the cell’s operation potential and electric ground on both sides.
- There is an isolation between the fuel cell model and its surroundings.

### Mesh analysis for the model

**Fig. 5:**
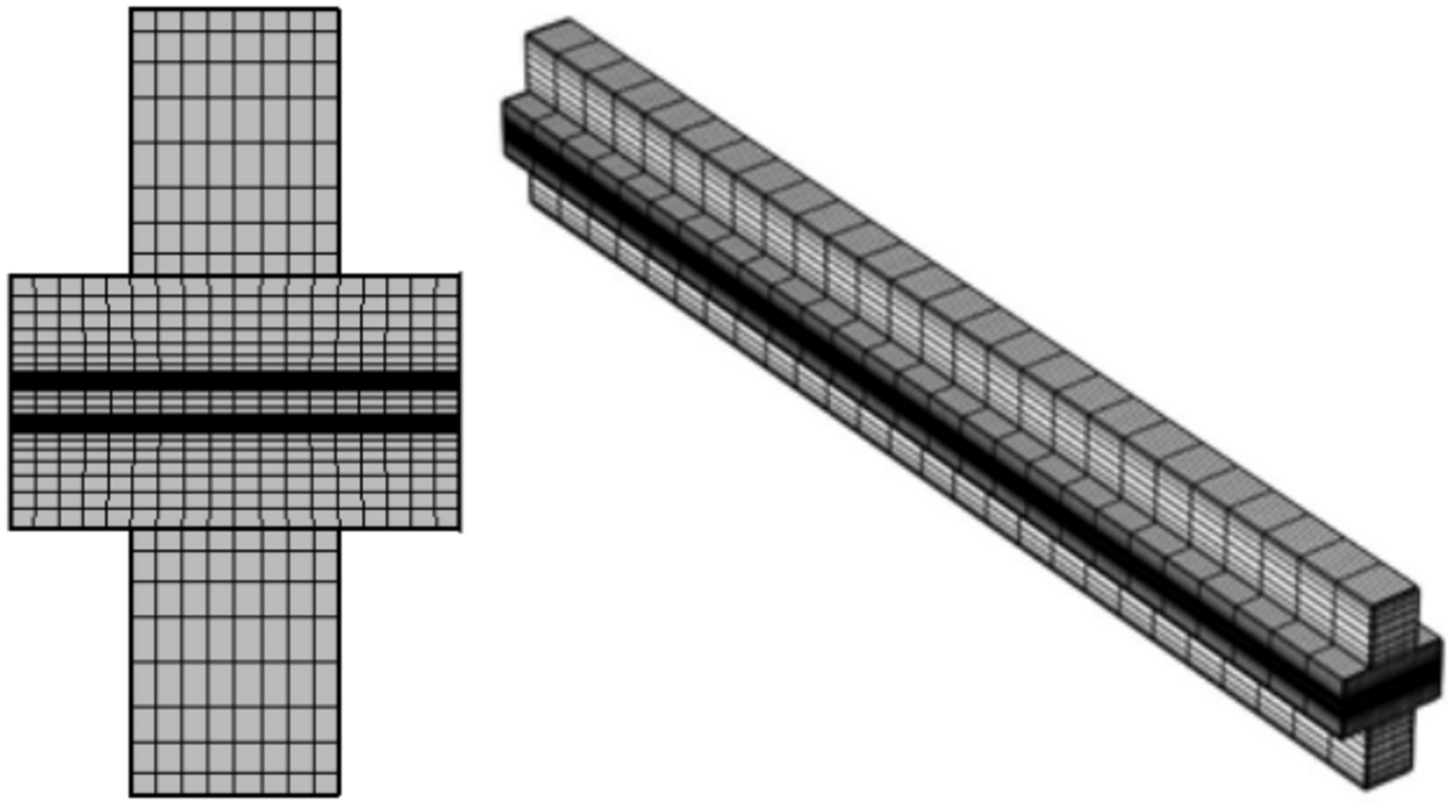
The mesh analysis of the 3D model of the HT_PEMFC.

As illustrated by the mesh analysis of the 3D model in Fig. 5, one case is done based on the finite element method, which is done for all cases of study. In each case before computing the study results, mesh analysis is done for the cases. Parameters that characterize the meshing specifics of this study, the statistics and the settings of the mesh model are described in Table 2.

**Table 2:** Mesh statistics of the 3D model of the HT_PEMFC.

Description	Value
Minimum element quality	$0.001619$
Average element quality	$0.03265$
Hexahedral elements	$19708$
Quadrilateral elements	$7392$
Edge elements	$972$
Minimum element size	$3.6\times {10}^{-4}$
Curvature factor	$0.6$
Resolution of narrow regions	$0.5$
Maximum element growth rate	$1.5$

The smallest and largest element sizes are set to $0.36 \mu m$ and $0.002 mm$, respectively, to ensure precise model detail calculations. The thickness of the membrane layer, which consists of three meshing elements, has a complicated and important impact on the fuel cell’s performance.

## Results and discussions

### Accuracy of model

To determine the model accuracy, an experimental consequence from Ref.^56^ is designated to be compared with the results of the simulated model. The polarization curve of the I-V relation started from $450 mV$ to $1000 mV$, where the model input is voltage and obtained the current. The temperature in the simulation was set as 180 ºC and the open circuit voltages in the experiment are $930 mV$, due to the same operational conditions as the data of the experiment.

**Fig. 6:**
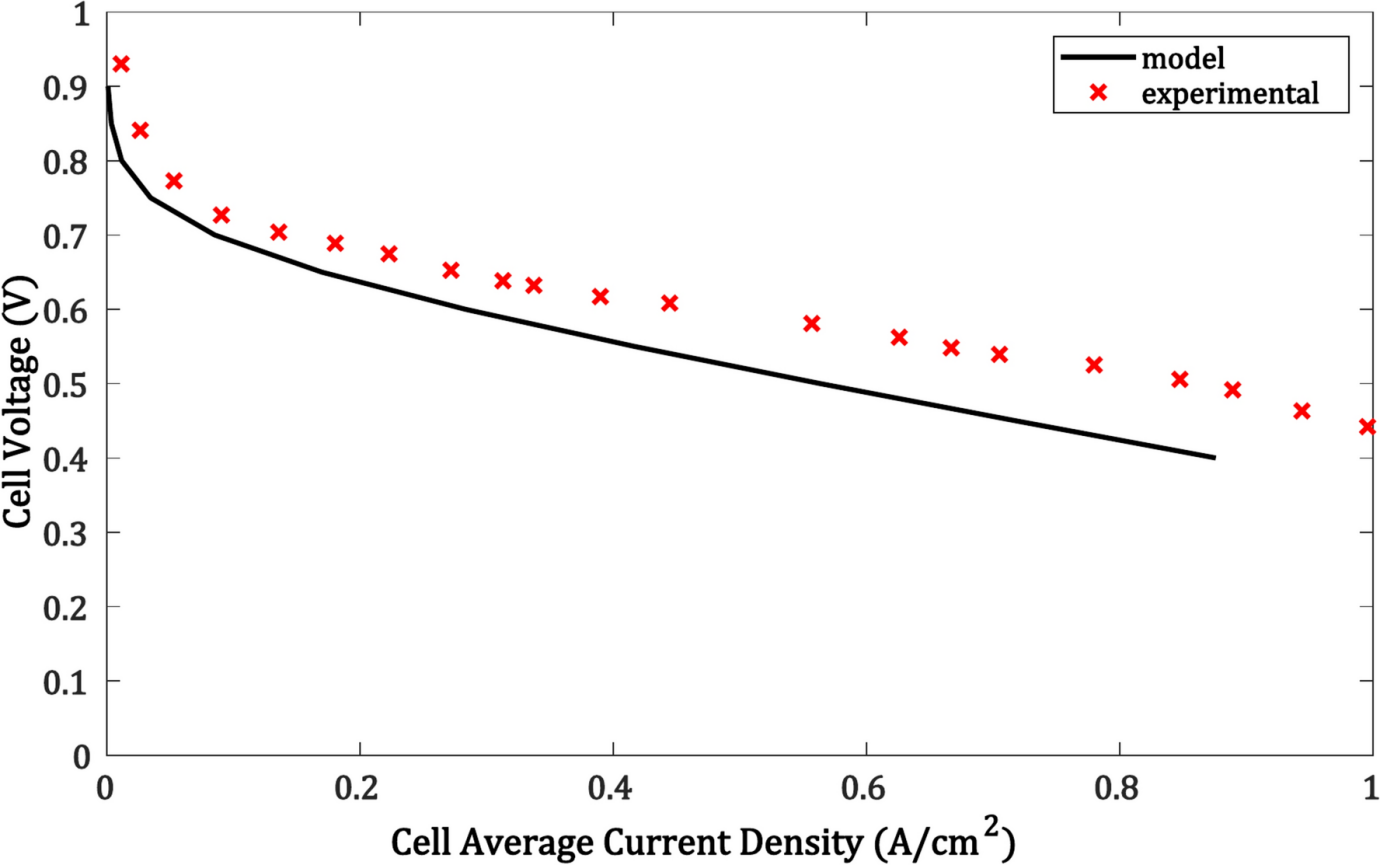
Comparison between experimental model and simulation model.

The simulation findings are lower than the experimental OCV, as shown in Fig. 6. This discrepancy could be attributed to several fuel cell parameters, including temperature, fuel distribution, and compressing force. According to the optimal voltage output equation, the OCV may drop slightly as the temperature rises. Consistent with the experiment settings, the membrane thickness is fixed at 100 µm. Based on Fig. 6, it can be inferred that the simulation and experimental findings behave similarly and have the closest values. The error between the two results is negligible for accepted values within the range of (0.01241 − 0.039) V in the low current density area.

The differences may be summed up as follows: First, the fuel cell’s performance in the simulation is superior to its experimental performance in the low current density area because the activation loss in the experiment test has a value that is more significant than the results of the simulation model.

Given that gas isn’t optimal in real life. The characteristics of the input gas are fixed in this model. The experiment’s characteristics could, however, differ. The model fuel cell’s porous medium material is said to have certain characteristics that are faultless. Nevertheless, there are always some shortcomings in the experiment’s materials. Even yet, the experimental test is not performed from an open circuit voltage of 0 V. Because of the fuel cell’s safety precautions, the voltage may drop quickly at very low voltages owing to concentration loss.

### Effect of parameters on the performance

#### Effect of membrane thickness

**Fig. 7:**
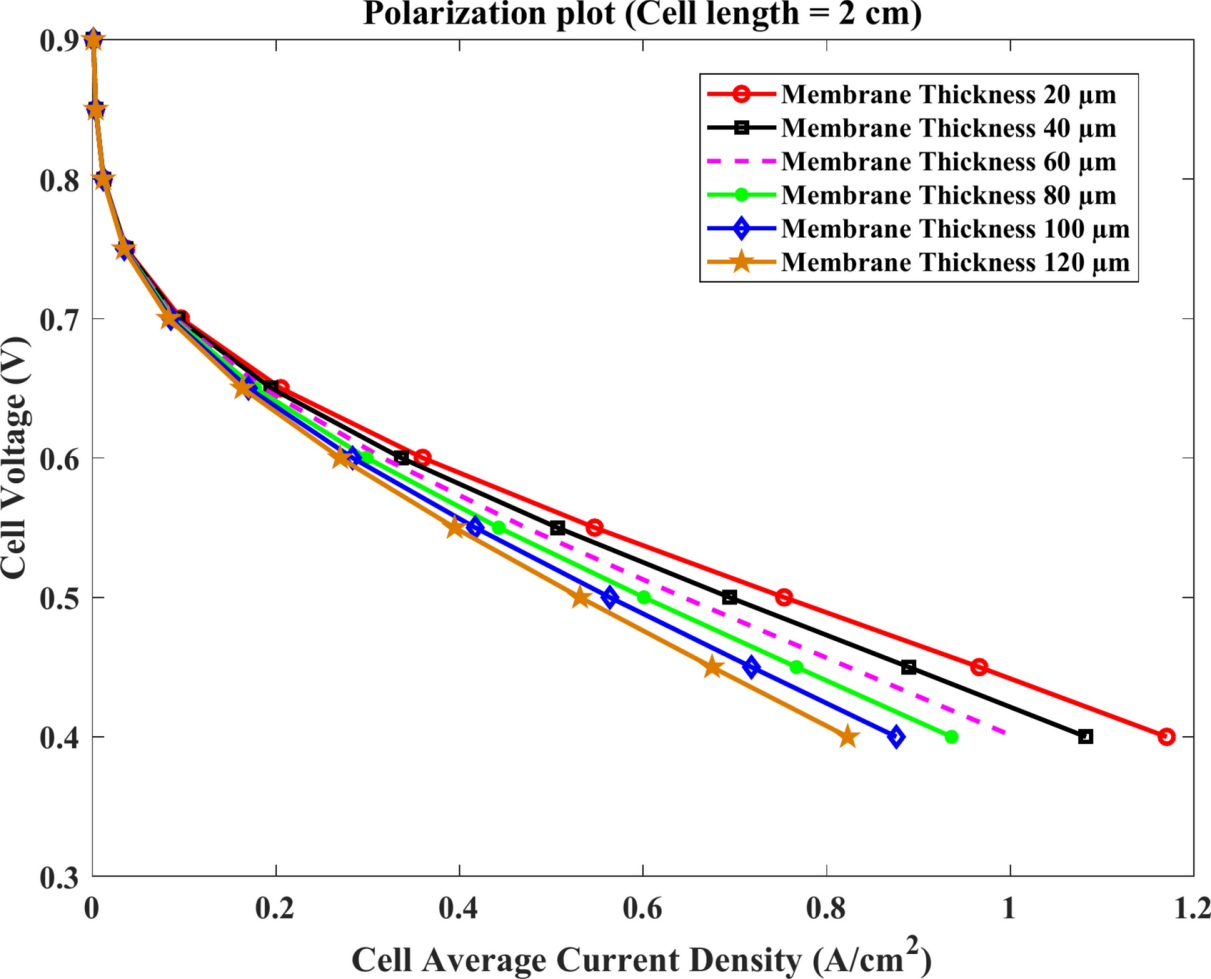
Polarization curve of cell length $=2\text{ cm}$.

**Fig. 8:**
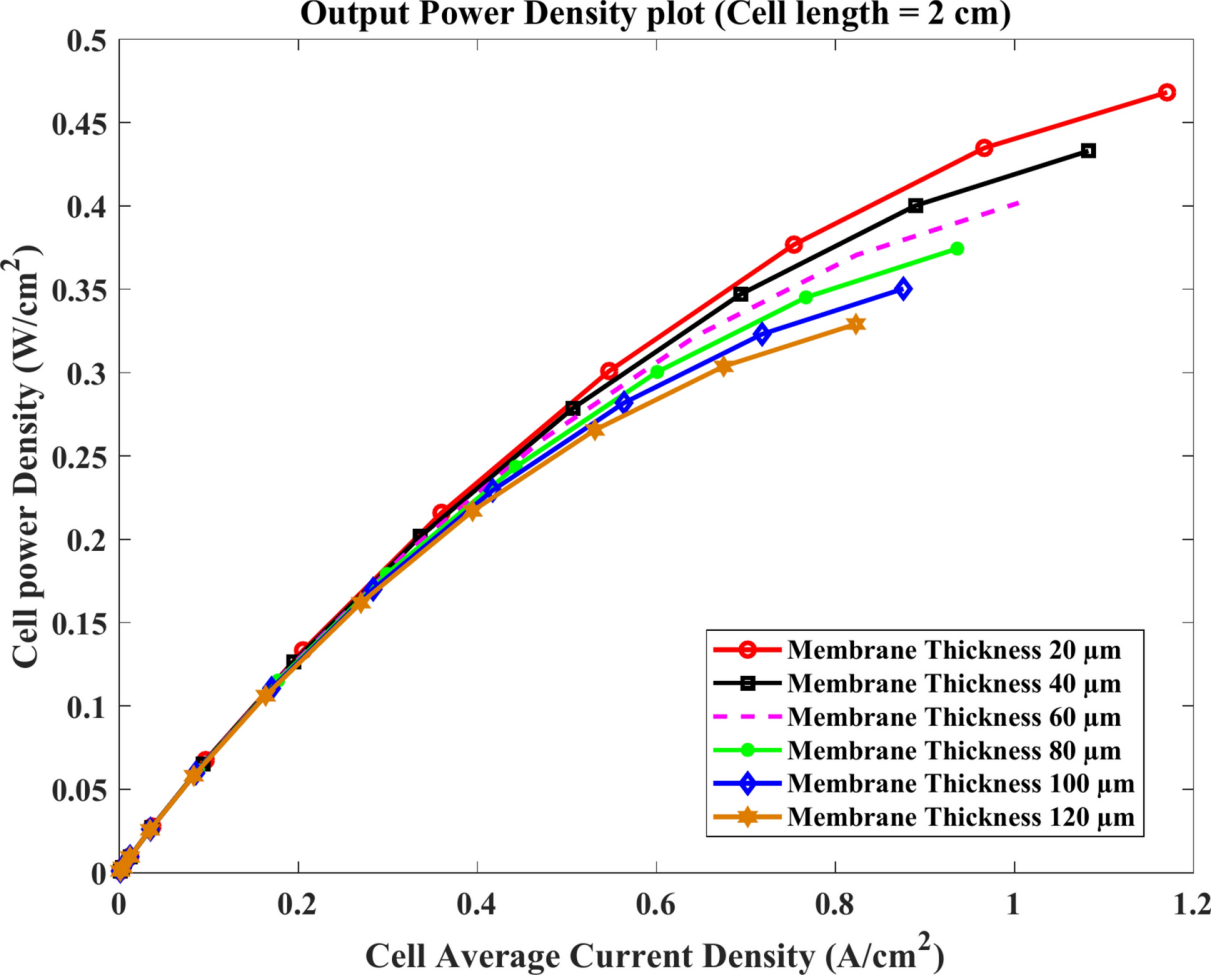
Cell power density(W/cm^2^) cell length $=2\text{ cm}$.

**Fig. 9:**
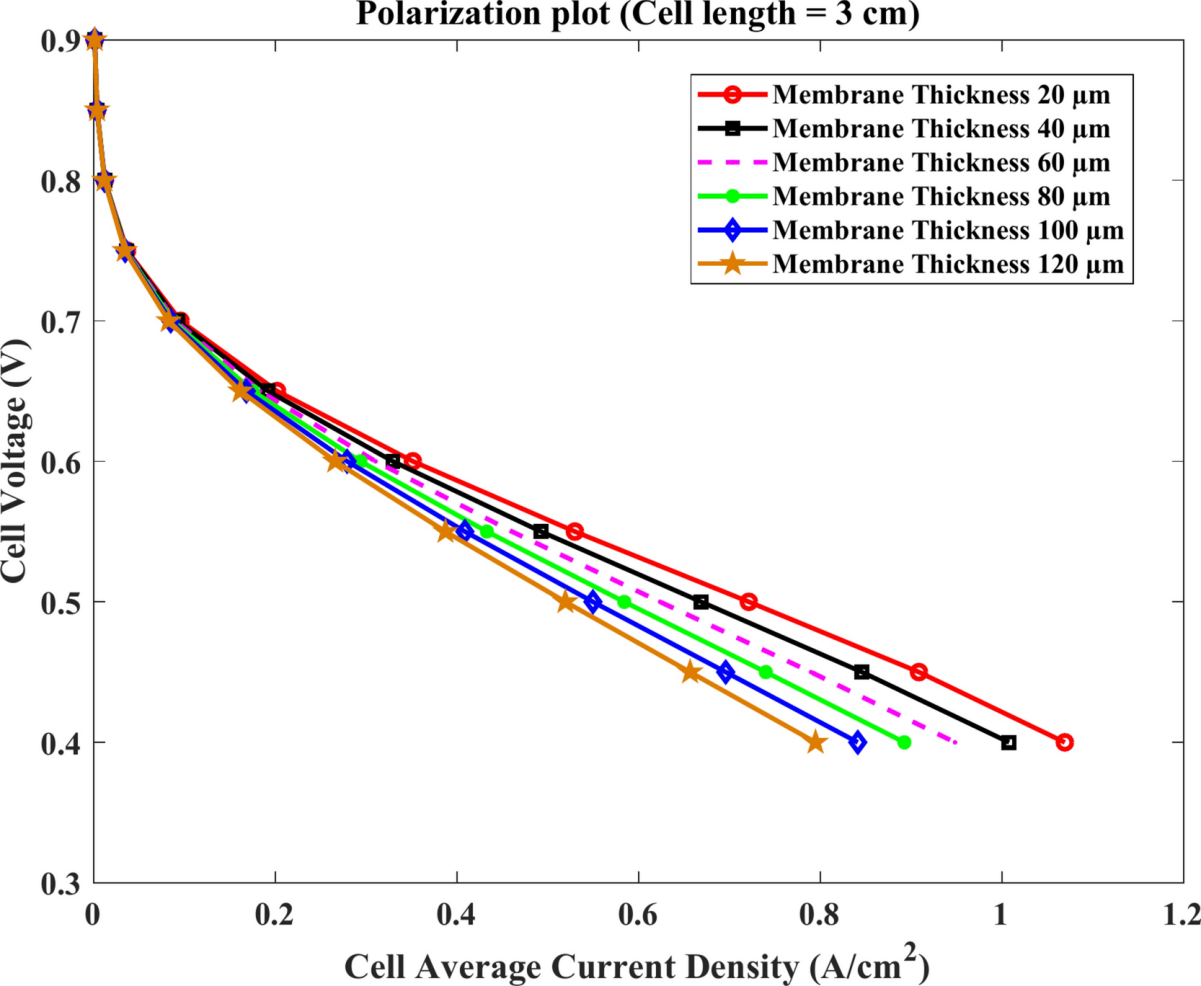
Polarization curve of cell length $=3\text{ cm}$.

**Fig. 10:**
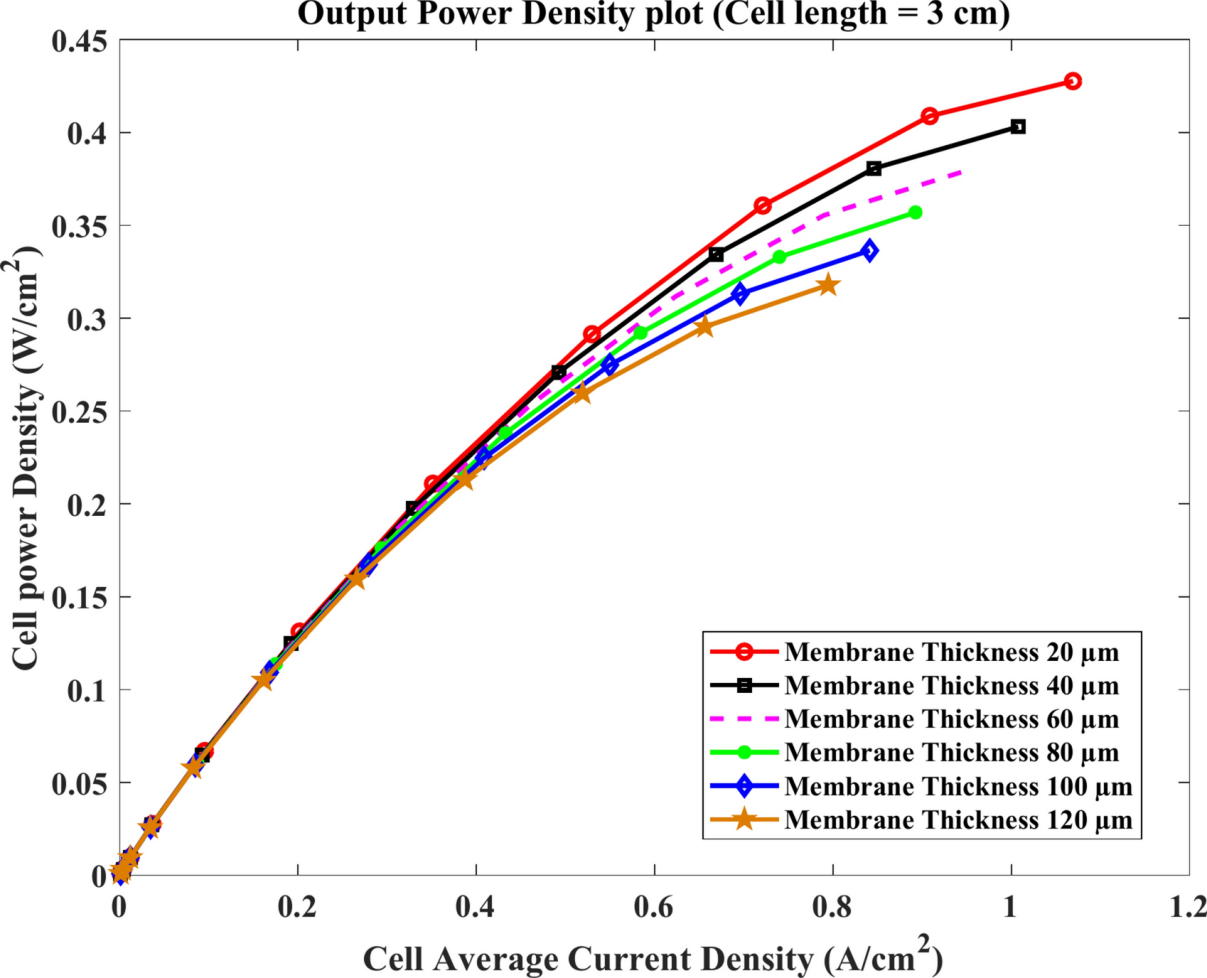
Cell power density (W.cm^-2^) of cell length $=3\text{ cm}$.

**Fig. 11:**
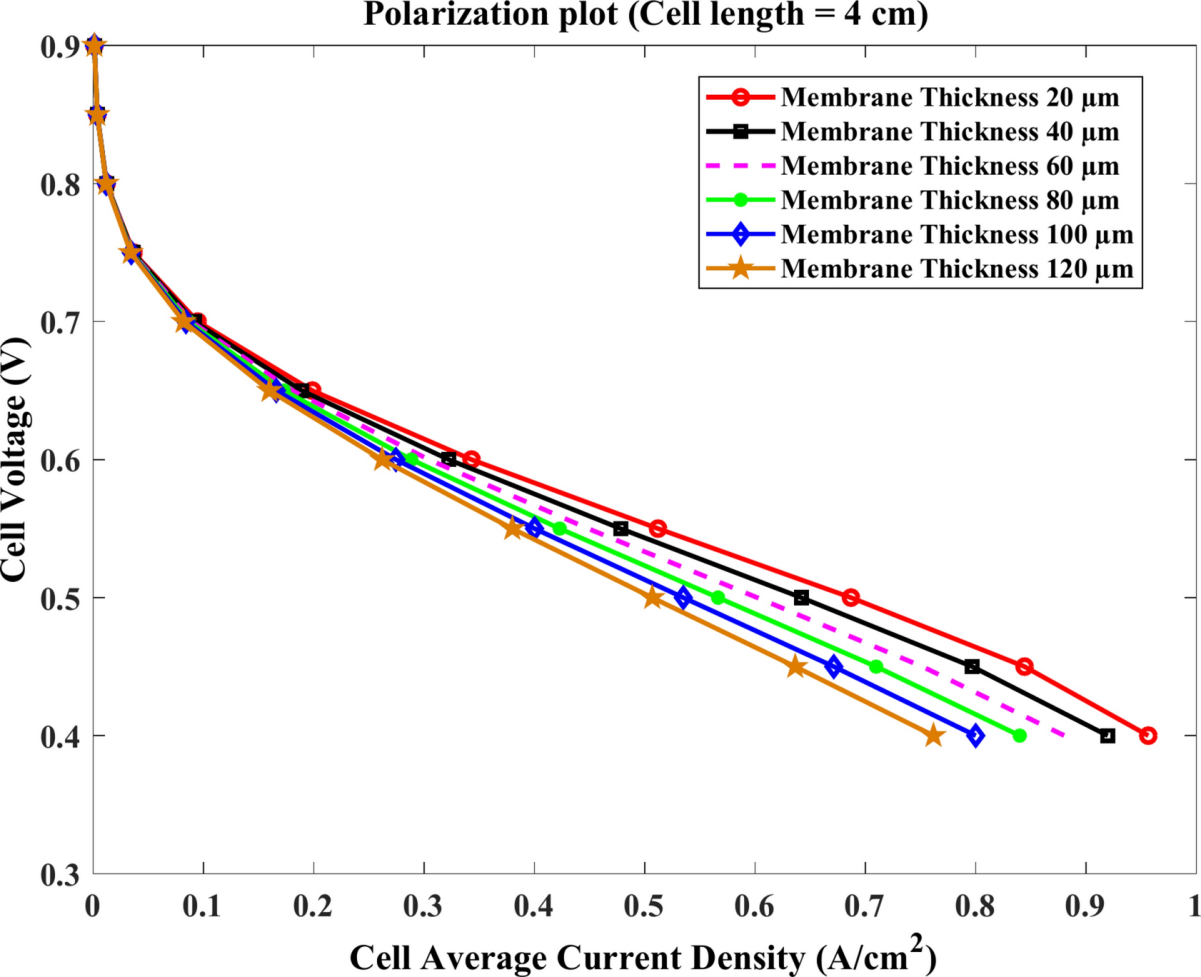
Polarization curve of cell length $=4\text{ cm}$.

**Fig. 12:**
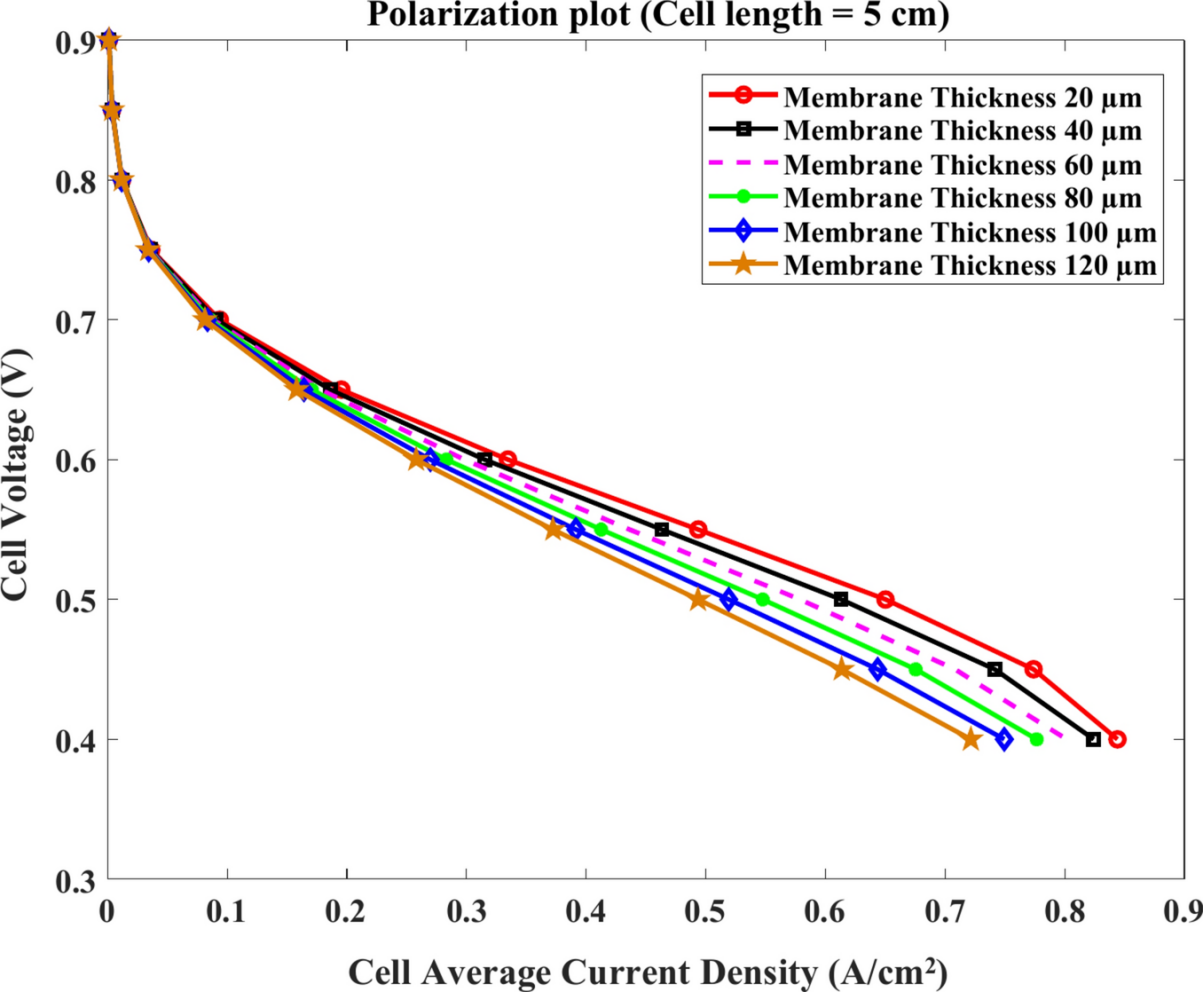
Polarization curve of cell length $=5 cm$.

**Fig. 13:**
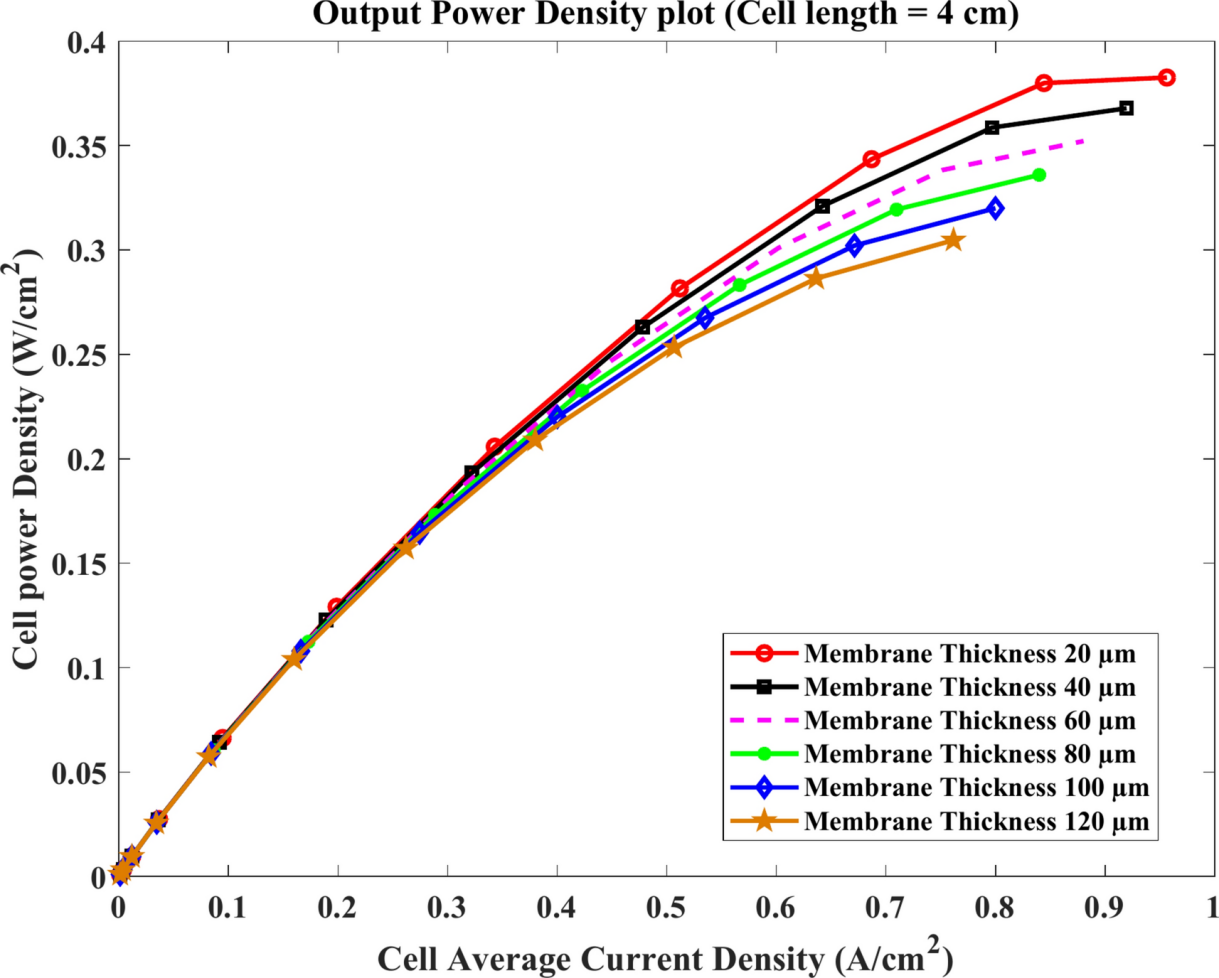
Cell power density ($\text{W}.{\text{cm}}^{-2}$) of cell length $=4\text{ cm}$.

**Fig. 14:**
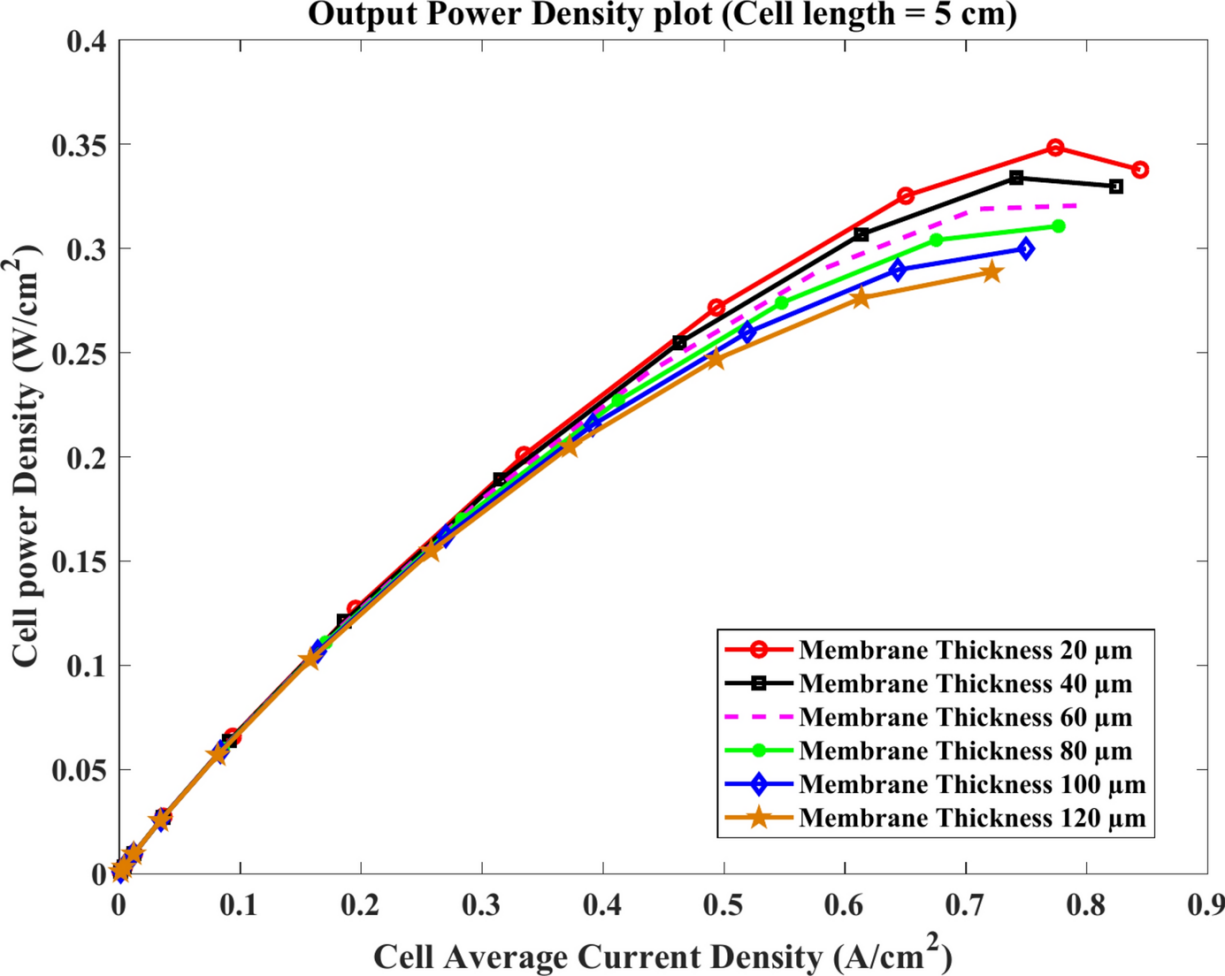
Cell power density ($\text{W}.{\text{cm}}^{-2}$) of cell length $=5 cm$.

According to studies, the membrane’s thickness can be adjusted between 20 and 120 µm^57–59^. Thus, at 180º C and 20 mm in length, the thicknesses of 20 µm, 40 µm, 60 µm, 80 µm, 100 µm, and 120 µm are tested. The results displayed in Figs. (7–14) show that the performance of HTPEMFC diminishes as membrane thickness increases. In specifics, the membrane thickness may alter the ohmic loss and concentrate loss in the I-V curves but does not affect the open circuit voltage. As the current increases from 0 A to $1.2\text{ A}.{\text{cm}}^{-2}$, the gaps between the curves widen.

Figures 7 and 8 show the polarization and the cell power density curve, respectively, for all models with a thickness of 20 µm to 120 µm by increasing 20 µm, where the length of the cell is 2 cm, and the temperature is 180 °C. The outcomes in Fig. 7 demonstrate that the performance of HTPEMFC increases as membrane thickness reduces, while the maximum values of power density are $(0.402$, $0.445$ and $0.483)\text{ W}.{\text{cm}}^{-2}$ at $60$μm, $40$ μm and 20 μm thickness, respectively, according to Fig. 8. In addition, the minimum power density occurs at the maximum membrane thickness 120 μm. This is at $2\text{ cm}$ of the fuel cell length where ohmic losses are less.

The shown results point out that the HTPEMFC performance decreases with the rise of membrane thickness. The maximum current density of $1.2\text{ A}.{\text{cm}}^{-2}$ at the model of membrane thickness $20$ μm and also the maximum power density output is $0.483\text{ W}.{\text{cm}}^{-2}$ for the same thickness.

Furthermore, Figs. 9 & 10 indicate the polarization and the cell power density curve at the cell length $3\text{ cm}$ with different thickness. The results reveal that the maximum current density is about $1.12\text{ A}.{\text{cm}}^{-2}$ at the model of membrane thickness $20$ μm and the producing voltage of HTPEMFC declines with the increase of membrane thickness. Also, the maximum power density output is given $0.43\text{ W}.{\text{cm}}^{-2}$ for the same model.

Similarly, the voltage of the cell approximately decreases at the same rate with the current at the membrane length of $4\text{ cm}$ and $5\text{ cm}$ for the same model thicknesses as shown clearly in Figs. 11 and 12. For $4\text{ cm}$ length, the value of the current density at $0.4\text{ V}$ equals about $0.97$, $0.88$, and $0.77 \text{A}.{\text{cm}}^{-2}$ at $20$, $60$ and $120$ μm, respectively. Compared to $5\text{ cm}$, the current falls to $0.85$, $0.8$, and $0.71\text{ A}.{\text{cm}}^{-2}$ at the same conditions.

In contrast, it is clear that the power density is directly proportional to the current as illustrated in Figs. 13 and 14. In general, the increase of power density continues with the rise of the current for all used thickness according to the last-mentioned figures. However, Fig. 12 shows that the maximum value of the power density has occurred on the current lower than its maximum. Relating to 20 µm thickness, the peak of power density output is $0.355\text{ W}.{\text{cm}}^{-2}$ at $0.78\text{ A}.{\text{cm}}^{-2}$ while the current density reached $0.87\text{ A}.{\text{cm}}^{-2}$.

These results are significantly consistent with the result of LTPEMFC introduced by ref.^60^. As soon as the LTPEMFC drives by using pure hydrogen, the fuel cell performance becomes better with the thinner membrane due to the lowest conductivity resistance of ion^60,61^. Relating to HTPEMFC, the main conductive ions are the phosphoric acid monocular rather than the PBI membrane. Within the PBI framework, there are many monocular phosphoric acids^62^. Furthermore, there may be reduced internal resistance in HTPEMFCs with thinner membranes. Nevertheless, the PBI membrane must possess adequate mechanical strength to maintain the redox reaction at high temperatures; therefore, the membrane cannot be very thin. Moreover, the performance degradation of the Nafion membrane is investigated by using different thicknesses^63^.

Although the results show that a thinner membrane has better performance, under degradation tests, thinner membranes are easier to fail due to their worse structural reliability consistent with^60–64^. For that, the appropriate thickness of membrane is from 20 to 60 µm. Practically, the easily obtained PBI membrane thickness is approximately 40 µm^65^.

#### Two dimension models result

**Fig. 15:**
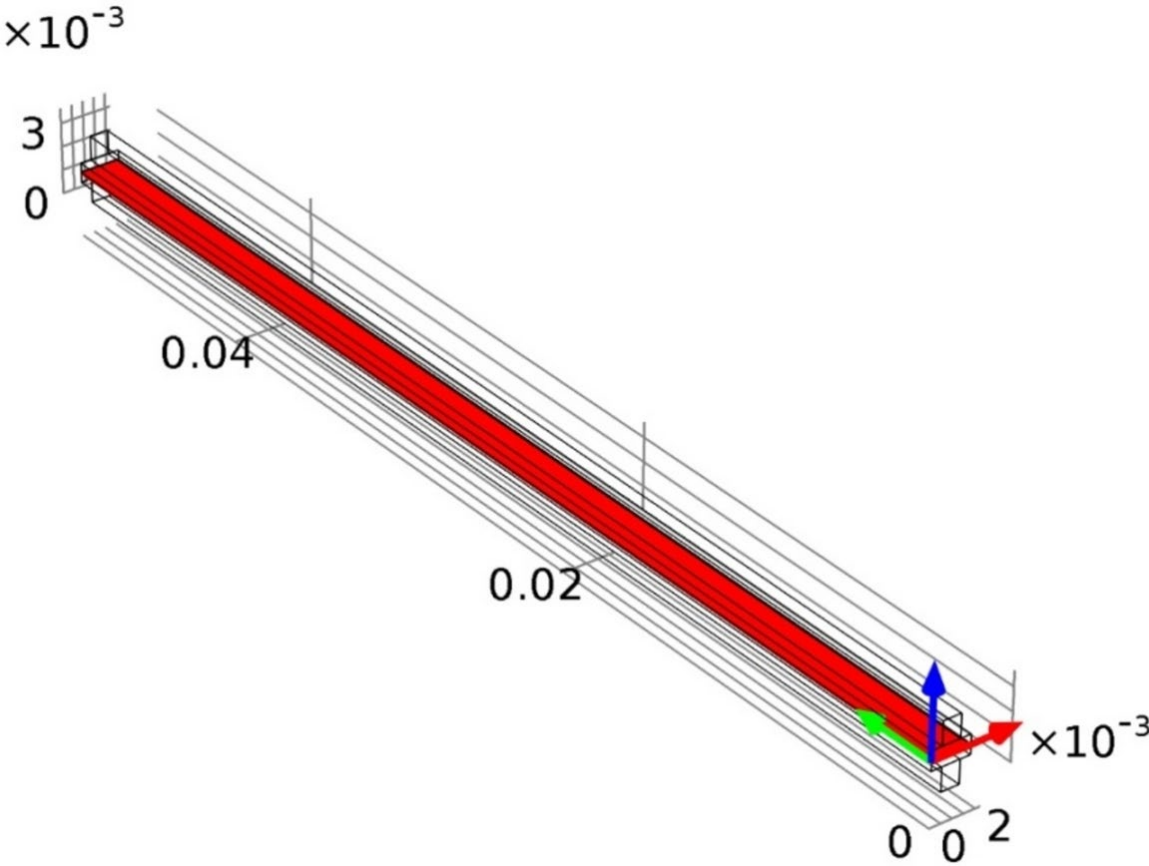
A cut plane in the membrane layer.

Also, two-dimension model results are used to obtain the current distribution through the fuel cell and the thickness effect on its performance. Figure 15 shows a cut plane taken on the membrane layer where the electrolyte current density was studied.

**Fig. 16:**
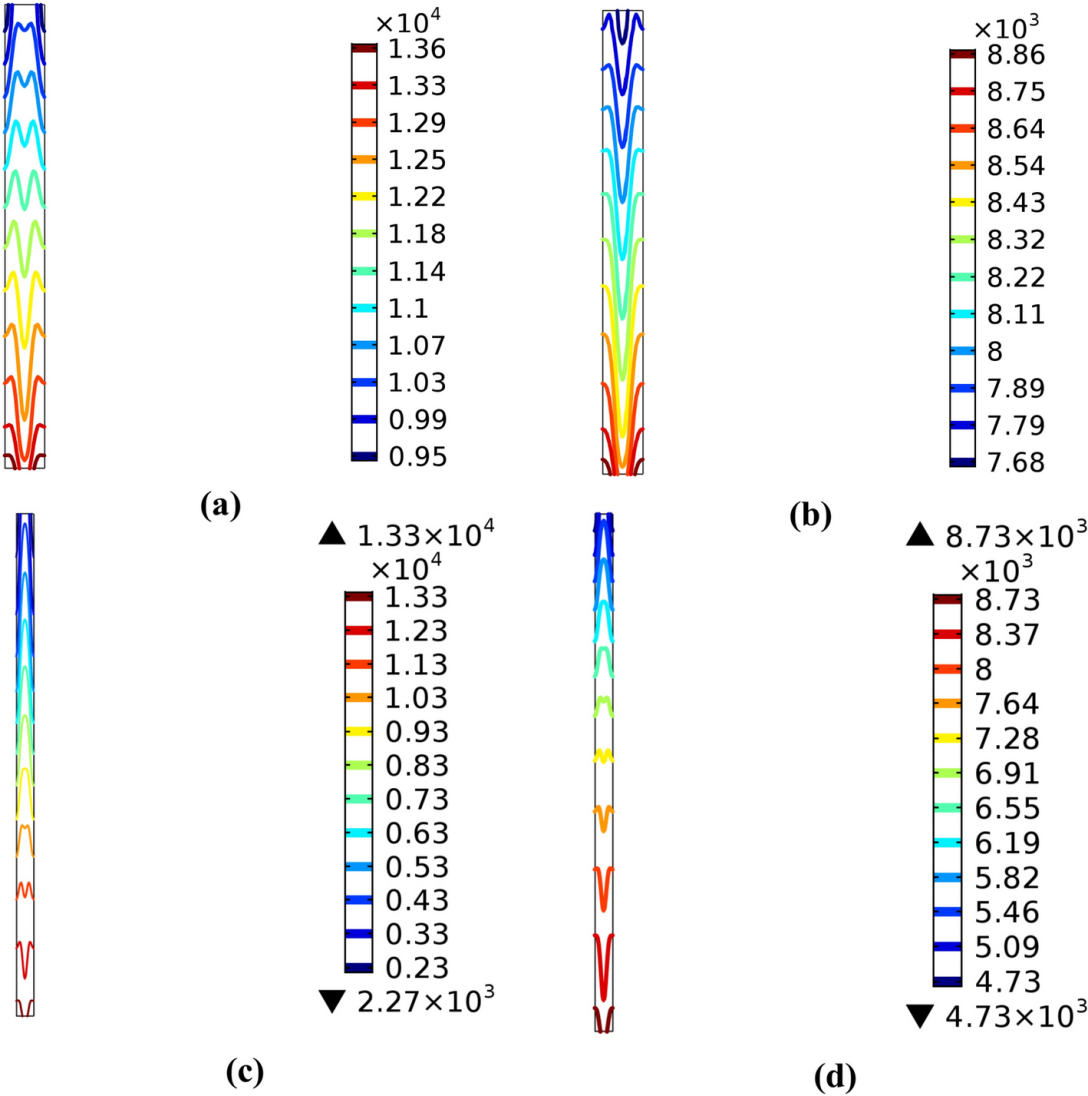
Electrolyte current density by $(\text{A}.{\text{cm}}^{-2})$ for different cell length and membrane thickness (**a**) 2 cm and 20 μm, (**b**) 2 cm and 120 μm, (**c**) 5 cm and 20 μm, and (**d**) 5 cm and 120 μm, respectively.

Figure 16 represents four case studies for two thicknesses $20$ and $120$ μm at two lengths $2$ and $5\text{ cm}$, alternately. According to this figure, with $2\text{ cm}$ thickness, the current density value rises from $0.95$ to $1.36\text{ A}.{\text{cm}}^{-2}$ at $20$ μm. In addition to $120$ μm, the maximum value of the current reaches $0.886 \text{A}.{\text{cm}}^{-2}$. For cases c & d, the length of fuel cell is $5\text{ cm}$ and the membrane thickness changed from $120$ μm to $20$ μm, it was found that the current density value increased from $0.227$ to $1.33\text{ A}.{\text{cm}}^{-2}$. From these values, we can find that the membrane thickness has a more effective influence on the current density produced from the fuel cell than the effect of the length of the fuel cell.

### Length effect of fuel cell

**Fig. 17:**
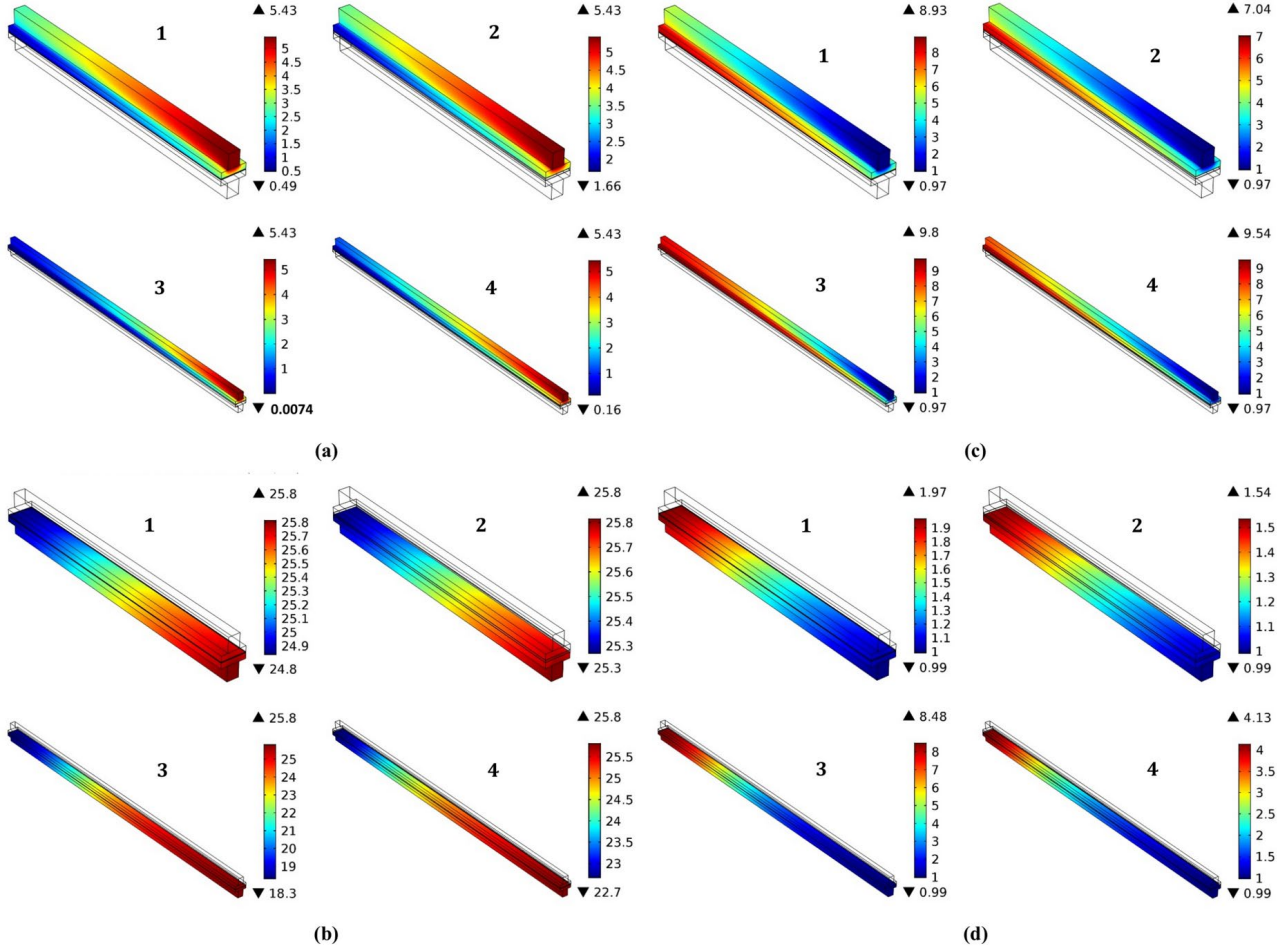
By $(\text{mol}.{\text{m}}^{-3})$ the surface concentration (**a**) Oxygen (**b**) Hydrogen (**c**) cathode water and (**d**) anode water at different geometrical parameters, where the cell length and membrane thickness are (2 cm & 20 μm) in 1 and 2 figures, and (5 cm & 120 μm) in 3 and 4 figures, respectively.

Both the cathode and anode catalyst layers are involved in the thickness of this model. In 3D, Fig. 17a to d show the surface molar concentration of hydrogen, oxygen and water at each side of the anode and cathode in the different cases of study of the fuel cell model with different thicknesses of 20 µm and 120 µm, and various lengths of the fuel cells 2 cm and 5 cm. The operating temperature was set as $180^\circ{\rm C}$ and the thickness of the catalyst layer was fixed.

Figure 17a shows the surface molar of oxygen concentration at the cathode layer as it has a value around $5.4 (\text{mol}.{\text{m}}^{-3})$ for the four cases of study that clear that this concentration is unaffected by varying the parameters of fuel cell length or the thickness of the membrane. Similarly, Fig. 17b demonstrates the surface molar of hydrogen concentration at the anode layer of the fuel cell as it has a value from $25$ to $25.8 (\text{mol}.{\text{m}}^{-3})$ for the four cases of study that clear that this concentration of hydrogen is unaffected by changing the parameters of fuel cell length or the thickness of the membrane.

Figure 17c reveals the surface molar concentration of water at the cathode side has a value changed from $7.04\text{ mol}.{\text{m}}^{-3}$ for fuel cell that has a length of 2 cm and a membrane thickness of 120 µm, $8.93\text{ mol}.{\text{m}}^{-3}$ for fuel cell that has a length of 2 cm and membrane thickness 20 µm and a value of $9.54\text{ mol}.{\text{m}}^{-3}$ for fuel cell that has a length of 5 cm and a membrane thickness of 120 µm and a value of $9.8 \text{mol}.{\text{m}}^{-3}$ for fuel cell that has a length of 5 cm and a membrane thickness of 20 µm.

Figure 17d shows the surface molar concentration of water at the anode side has a value changed from $1.54 \text{mol}.{\text{m}}^{-3}$ for fuel cell that has a length of $2\text{ cm}$ and a membrane thickness of $120$ µm to a value of $1.97 \text{mol}{.\text{m}}^{-3}$ for fuel cell that has a length of $2\text{ cm}$ and a membrane thickness of 20 µm and a value of $4.13 \text{mol}.{\text{m}}^{-3}$ for fuel cell that has a length of $5\text{ cm}$ and a membrane thickness of $120$ µm to a value of $8.48 \text{mol}.{\text{m}}^{-3}$ for fuel cell that has a length of $5\text{ cm}$ and a membrane thickness of $20$μm. Accordingly, it is clear that this concentration of water at the cathode and anode side of the fuel cell is highly affected by varying the fuel cell length more than the change of its membrane thickness.

The effect of fuel cell length influences on cell performance output due to its ohmic polarization and losses^66^. The previous results indicate that the cathode water concentration and the anode water concentration rise from $7$ to $9 \text{mol}.{\text{m}}^{-3}$ with the increase in fuel cell length from 2 to 5 cm corresponding to the output in^67^. In addition, the increase of length in the fuel cell increases the ohmic resistance of both the ionic and electronic conductivity, which leads to lower performance. As it can be observed that the optimal length of a fuel cell could be about 20–40 mm consistent with^68,69^.

#### Membrane conductivity

**Fig. 18:**
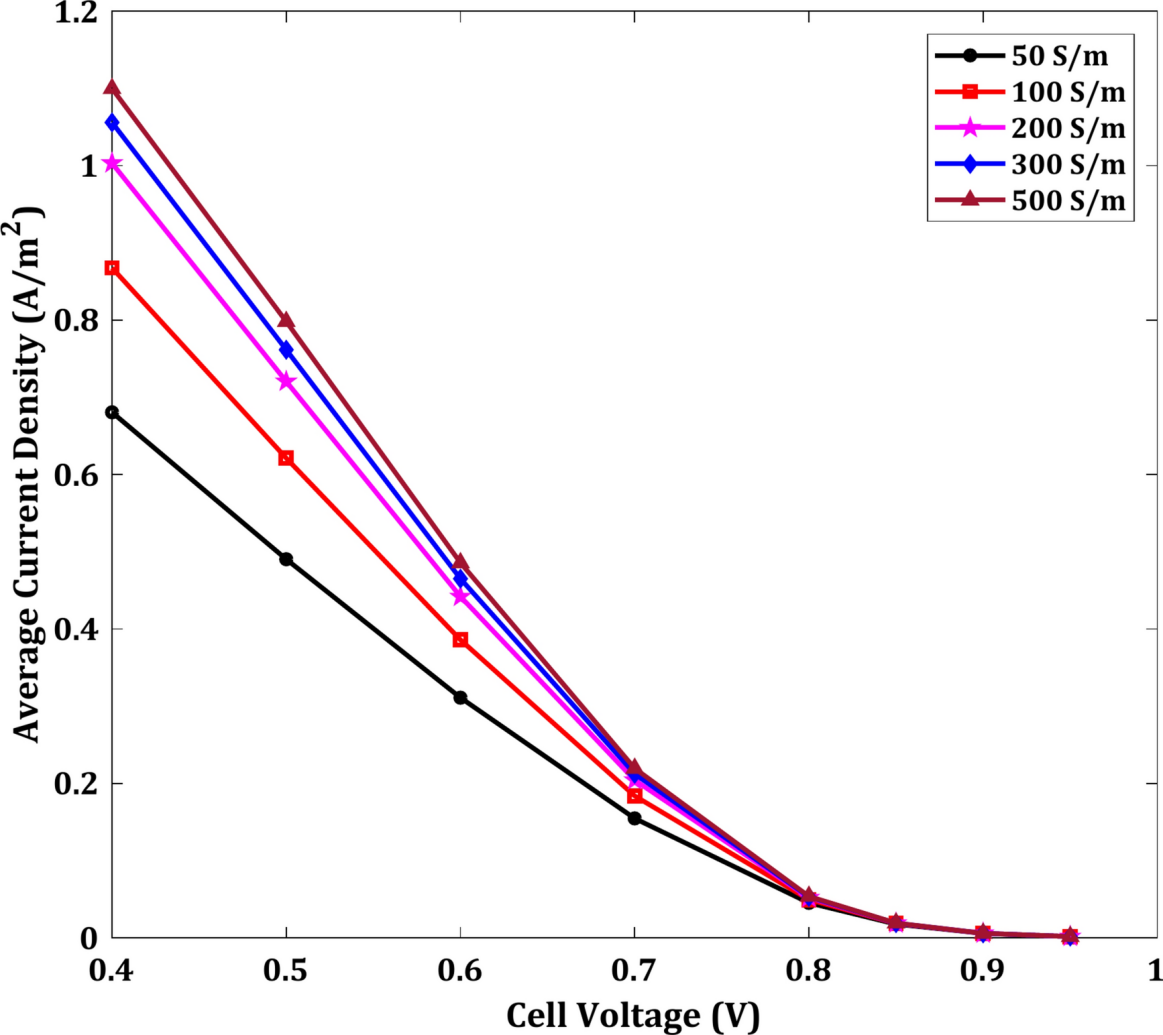
Average current density at different conductivity.

In this section, the result of the effect of conductivity on the current density is presented with the difference in cell voltage. It was found that there is a significant effect of the conductivity value at smaller cell voltage, as shown in Fig. 18. The whale of curves in this figure are at a constant fuel cell length and membrane thickness 2 cm and $100 \mu m$, respectively. At 0.4 V cell voltage, the increase of conductivity from 50 S/m to 100 S/m raises the current density by 28%. But this percentage approximately decreases to 2% at 0.9 V cell voltage. Therefore, the influence of the conductivity parameter is more evident at low cell voltage values.

## Conclusion

In conclusion, this study presents a simulation technique for analyzing a 3D model of HTPEMFC, focusing on its operational principles and reaction mechanisms. Through finite element method analysis, the study investigates the effects of key parameters, particularly the proton exchange membrane thickness and fuel cell length, on its performance. Then, the results of the simulation model can be summarized in the following points.

- The model performance of HTPEMFC is enhanced by the decrease of membrane thickness, where the maximum power density is $0.402\text{ W}.{\text{cm}}^{-2}$, and $0.483\text{ W}.{\text{cm}}^{-2}$ at the thickness of $60$ μm, and $20$ μm, respectively.
- The maximum current density is about $1.12\text{ A}.{\text{cm}}^{-2}$ at the model of membrane thickness $20$ μm.
- The thickness of the membrane has a more effective influence on the current density produced from the fuel cell than the effect of the length of the fuel cell.
- The results demonstrate the feasibility of utilizing the analytical model to optimize single PEM fuel cell performance.
- The effect of conductivity parameter is more obvious at low voltage values.
- These results promise to inform the operation and design of HTPEMFCs in real-world applications.

These results can be developed the current simulation model to include other factors affecting fuel cell performance, such as temperature and humidity distribution, and the effect of degradation on long-term performance. Furthermore, the model can be used to optimize the values of the different fuel cell parameters and enhance the performance of the fuel cell system.

## Data Availability

All data generated or analysed during this study are included in this published article.
